# Effects of Glomalin-Related Soil Protein Driven by Root on Forest Soil Aggregate Stability and Carbon Sequestration during Urbanization in Nanchang, China

**DOI:** 10.3390/plants12091847

**Published:** 2023-04-30

**Authors:** Changyongming Cai, Fei Huang, Yaying Yang, Suqin Yu, Sujia Wang, Yulu Fan, Qiong Wang, Wei Liu

**Affiliations:** 1 Jiangxi Provincial Key Laboratory of Silviculture, Jiangxi Agricultural University, Nanchang 330045, China; yming8023@163.com (C.C.); hf20000206@163.com (F.H.); yangyy@stu.jxau.edu.cn (Y.Y.); ysq202248@163.com (S.Y.); 15797747054@163.com (S.W.); 15079093278@139.com (Y.F.); 2 College of Forestry, Jiangxi Agricultural University, Nanchang 330045, China

**Keywords:** glomalin-related soil protein, soil aggregate stability, urbanization intensity, soil organic carbon

## Abstract

Glomalin-related soil protein (GRSP) is a hydrophobic protein released by arbuscular mycorrhizal fungi. It is an important component of the soil carbon pool, and it improves the soil aggregate structure; however, it remains unclear whether GRSP can enhance soil carbon sequestration and improve soil quality during rapid urbanization. The built-up area in Nanchang, China was the study area, and the proportion of impervious surface area was the parameter of urbanization intensity. A total of 184 plots (400 m^2^) were set up to collect soil samples (0–20 cm) for analysis. Aggregates of five particle sizes were sieved, and the percentage amounts of soil organic carbon (SOC) and GRSP for them were determined. The results showed that the easily extractable GRSP (EE-GRSP) and total GRSP (T-GRSP) contents of the four aggregates of <2 mm were 22–46% higher in low urbanization areas than those in high urbanization areas (*p* < 0.05), indicating that the higher urbanization intensity was associated with the lower GRSP content of different aggregates. The GRSP was significantly positively correlated with SOC (*p* < 0.05). Moreover, the contribution of GRSP to the SOC pool in the <0.25 mm aggregate was significantly higher than that in other aggregates. In addition, the EE-GRSP content was significantly positively correlated with mean weight diameter (MWD) and geometric mean diameter (GMD) in the four aggregates of <2 mm, whereas it was negatively correlated with fractal dimension (D) in the >2 mm, 1–2 mm and <0.053 mm aggregates. The T-GRSP content showed significant correlations only with MWD, GMD, and D in the 1–2 mm aggregate. This study revealed that increasing urbanization intensity can significantly reduce the GRSP content of different sized aggregates. Moreover, the GRSP content significantly promoted SOC sequestration, and the EE-GRSP content more significantly promoted soil aggregate stability than that of the T-GRSP. These findings provide new ideas for exploring the improvement of soil quality during the process of urbanization.

## 1. Introduction

Urbanization is an inevitable process in human social progress and development. Although urban areas account for only 1% of the global land area, they host more than 50% of the global population [1], and are continuing to expand rapidly [2]. The urban population is predicted to reach 67% of the total global population by 2025 [3], and thus urban area will be three times larger in 2030 than in 2000 [4]. With the rapid urbanization of China, rapid economic development will bring about unprecedented expansion of urban land and population [5,6]. China has mainly focused on old urban areas for urban expansion, which shows an obvious spatial distribution form of “urban–suburban–rural” [7]. Natural land has been gradually covered by gray infrastructure, such as roads and houses, and rapidly accelerating urbanization is bound to have a significant impact on urban soil ecosystems [8]. Soils are essential for human survival and development, providing important ecosystem services to humans, such as the regulation of water, carbon cycle, and greenhouse gas emissions [9]. However, severe human disturbances in cities have led to a general decline in urban soil quality, such as higher electrical conductivity, higher bulk density and compaction, lower porosity and infiltration rates, and poorer aeration and water storage capacity. All these factors degrade the soil structure [10]. Moreover, urban soil sealing can cause a significant decrease in the soil organic carbon (SOC) content, which affects the urban ecosystem carbon cycle and the global carbon cycle process, negatively impacting the soil carbon pool [2,11].

A beautiful city should be rooted in healthy soils; however, the current status of soil degradation often severely limits the healthy growth of plants. Therefore, a detailed study of changes that occur in soil structure and carbon content during urbanization is important in order to improve soil quality and promote soil carbon sequestration [12]. Soil aggregates are the basic units of the soil structure. The particle size composition and stability of soil aggregates are important evaluation indicators of soil structure [13], which affects soil porosity, erosion resistance, and nutrient cycling. Furthermore, this is an important basis for soil water and fertilizer conservation and supply functions [14]. In recent years, many studies have confirmed that soil aggregate stability has a significant positive correlation with SOC content [15,16]. On the one hand, soil aggregates are the main site of soil organic carbon fixation, which can protect SOC and weaken the decomposition and mineralization of microorganisms. On the other hand, SOC is an important binding material in forming soil aggregates. Its content level is the key to forming a good soil structure and improving soil resistance to oxygenolysis [17,18]. The formation of soil aggregates is influenced by several factors, such as plant root growth, changes in the soil physical and chemical properties (e.g., pH and bulk density), the activities of soil animals and microorganisms, and human disturbance. In particular, plant roots can effectively control soil erosion and thus stabilize soil structure, playing a crucial role in the formation of soil aggregates and sequestration of SOC [19]. However, the urban environment with its high human disturbance is more complex than the natural environment; therefore, it is necessary to study the changes in SOC and soil aggregate stability during the urbanization process.

Arbuscular mycorrhizal fungi (AMF) are widely distributed plant symbiotic organisms that form symbiotic relationships with the roots of more than 80% of higher plants [20]. The AM symbiont could significantly improve the ability of the host plant to absorb mineral nutrients (especially phosphorus) and enhance the ability of the host plant to resist disease, drought, and salt damage [21]. At the same time, AMF not only promote the formation of soil aggregates and the fixation of organic carbon, but also improve the ability of plants to absorb nutrients and withstand plant stress [22]. The AMF can spread through the soil through growth of the mycelium using a hydrophobic and stable protein called the glomalin-related soil protein (GRSP). It was first extracted successfully in 1996 by Wright et al. [23] at 121 ℃ with 20 mmol/L sodium citrate solution. The GRSP is classified into easily extractable GRSP (EE-GRSP) and total GRSP (T-GRSP) according to their extractability [24]. The GRSP content is an important indicator of soil quality [25]. First, GRSP enhances the stability of soil aggregates. Furthermore, GRSP is known as “super glue” because of its bonding ability, which is three to ten times stronger than that of common carbohydrates [24]. The GRSP can bond fine and unstable soil particles into larger and more stable aggregates, which improves the soil water permeability, soil stability, and porous structure [26], making soil organic matter less susceptible to decomposition and increasing SOC accumulation [27]. Second, GRSP is an important component of SOC and a major source of soil carbon pools [28]. The fixation of the SOC by GRSP plays an important role in ecological cycles, and the contribution of GRSP to the soil carbon pool is much larger than that of active microorganisms in the soil [29]. However, it is unclear how GRSP distribution during urbanization affects the structural stability of soil aggregates and soil carbon sequestration.

Nanchang is the capital city of Jiangxi Province in China, and is highly urbanized and its main soil type is red soil [30]. The population size reached 5.06 million at the end of 2021, and the proportion of impervious surface area was up to 78.64% (Nanchang Municipal Government, China). The special characteristics of red soil include it being acidic, impoverished, and cohesive. Inadequate and unreasonable exploitation has occurred during the rapid urbanization process. Increasingly compacted soils, reduced porosity, loss of organic matter, and the degradation of water and fertilizer storage capacity seriously affect the sustainability of the urban ecological environment [31]. Consequently, this study was undertaken to (1) analyze the distribution characteristics of the GRSP and SOC contents in different urbanization intensity areas in Nanchang city, (2) elucidate the contribution of GRSP to SOC, and (3) reveal the relationship between GRSP and soil structural stability. It was hypothesized that the distribution characteristics of GRSP would differ in soil aggregates from areas of different degrees of urbanization due to different levels of human interference. The results should benefit the evaluation of the effects of GRSP on soil aggregate stability and carbon sequestration during urbanization in Nanchang, China, and should help in providing a scientific basis and methods for the improvement of urban soil quality.

## 2. Results

### 2.1. Effect of the Urbanization Intensity on the Soil Aggregate Composition

Table 1 shows the differences in the percentage content of stable aggregates in soil water from areas of different urbanization intensities as indicated by the proportion of impervious surface area. The percentage values of the particle size range 0.053–0.25 mm were significantly lower at sites of low urbanization intensity than those from sites of high urbanization intensity (*p* < 0.05). Taking into consideration the stable aggregates distribution in water, regardless of the urbanization intensity, the 0.25–1 mm aggregates showed the highest values, while the >1 mm aggregates showed the lowest ones. Moreover, the values of the 0.25–1 mm aggregates were significantly higher than those of the aggregates > 1 mm (*p* < 0.05).

The differences in the stability of aggregates in water from areas of different urbanization intensities are shown in Table 2. Values of both the MWD and GMD indicators were the highest in samples from the low urbanization intensity region, whereas those of both the D and UAI indicators were the lowest in samples from the low urbanization intensity region, indicating that the aggregates in soil water were relatively stable in the samples from the low urbanization region.

### 2.2. Effect of Urbanization Intensity on SOC and GRSP in Different Soil Aggregates

**Figure 1:**
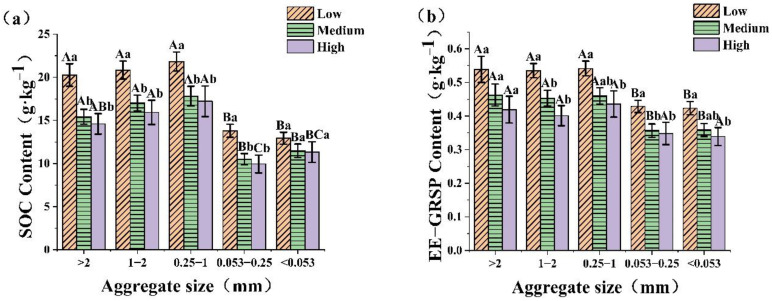

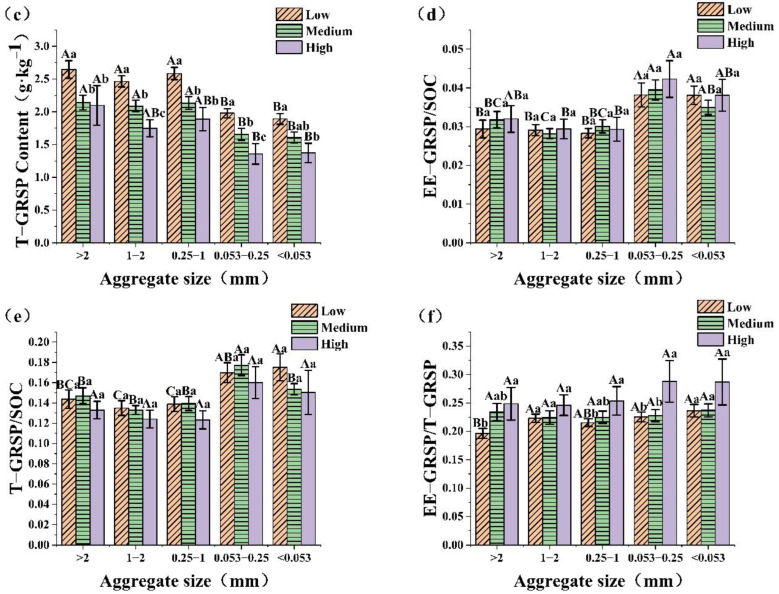
Characteristics of the GRSP and SOC contents of soil water stable aggregates with different urbanization intensities. Note: Different capital letters indicate significant differences among different aggregates at the same urbanization intensity (*p* < 0.05). Different lowercase letters indicate differences among different urbanization intensities in the same aggregate (*p* < 0.05). Soil organic carbon (SOC), easily extractable glomalin-related soil protein (EE-GRSP), total glomalin-related soil protein (T-GRSP). Effect of urbanization intensity on SOC (**a**), EE-GRSP (**b**), T-GRSP (**c**), EE-GRSP/SOC (**d**), T-GRSP/SOC (**e**), and EE-GRSP/T-GRSP (**f**) in different soil aggregates.

The distributions of SOC, EE-GRSP, and T-GRSP content in soil aggregates of different particle size are shown in Figure 1a–c. The SOC, EE-GRSP, and T-GRSP contents in most soil aggregates showed significant decreasing trends (*p* < 0.05) with an increasing urbanization intensity. However, only SOC in the <0.053 mm aggregate and EE-GRSP in the > 2 mm aggregate showed no significant difference among the different urbanization intensities. The T-GRSP in the five aggregates was significantly higher at a low urbanization intensity than at high urbanization intensity with 26–46%. Moreover, the SOC, EE-GRSP, and T-GRSP contents in the >0.25 mm particles were significantly higher than in the <0.25 mm particle sizes at low–medium urbanization intensities (*p* < 0.05).

There was no significant difference in the ratio of EE-GRSP/SOC and T-GRSP/SOC in soil aggregates with different urbanization intensities. However, for the same urbanization intensities (Figure 1d,e), the GRSP/SOC ratio of particle sizes >2 mm, 1–2 mm, and 0.25–1 mm was significantly lower than that of 0.053–0.25 mm and <0.053 mm (*p* < 0.05). The ratio of EE-GRSP/T-GRSP in the three particle sizes of >2 mm, 0.25–1 mm, and 0.053–0.25 mm showed a significant upward trend with the increase in urbanization intensity (*p* < 0.05) (Figure 1f). In the low urbanization intensity, the EE-GRSP/T-GRSP ratio in aggregates of >2 mm was significantly lower than that of 1–2 mm, 0.053–0.25 mm and <0.053 mm (*p* < 0.05).

### 2.3. Relationships between GRSP and SOC

**Figure 2:**
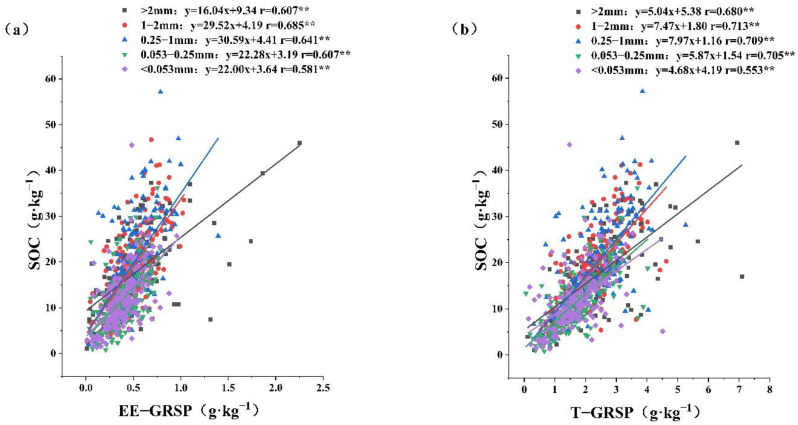
Correlations between SOC and GRSP in different aggregates. Note: *: *p* < 0.05, **: *p* < 0.01. Soil organic carbon (SOC), easily extractable glomalin-related soil protein (EE-GRSP), total glomalin-related soil protein (T-GRSP). Relationships between EE-GRSP and SOC (**a**), and between T-GRSP and SOC (**b**).

The significant correlations between GRSP (T-GRSP and EE-GRSP) and SOC in each aggregate type are shown in Figure 2 (*p* < 0.01). The strongest correlation was found in the 1–2 mm aggregate with the highest r value (0.685, 0.713), and the weakest correlation in the <0.053 mm aggregate with the lowest r value (0.581, 0.553). These results suggest that the GRSP content in the aggregates of larger particles promoted SOC storage more than that in the aggregates of smaller particles.

### 2.4. Relationships between GRSP and Soil Aggregate Stability

**Figure 3:**
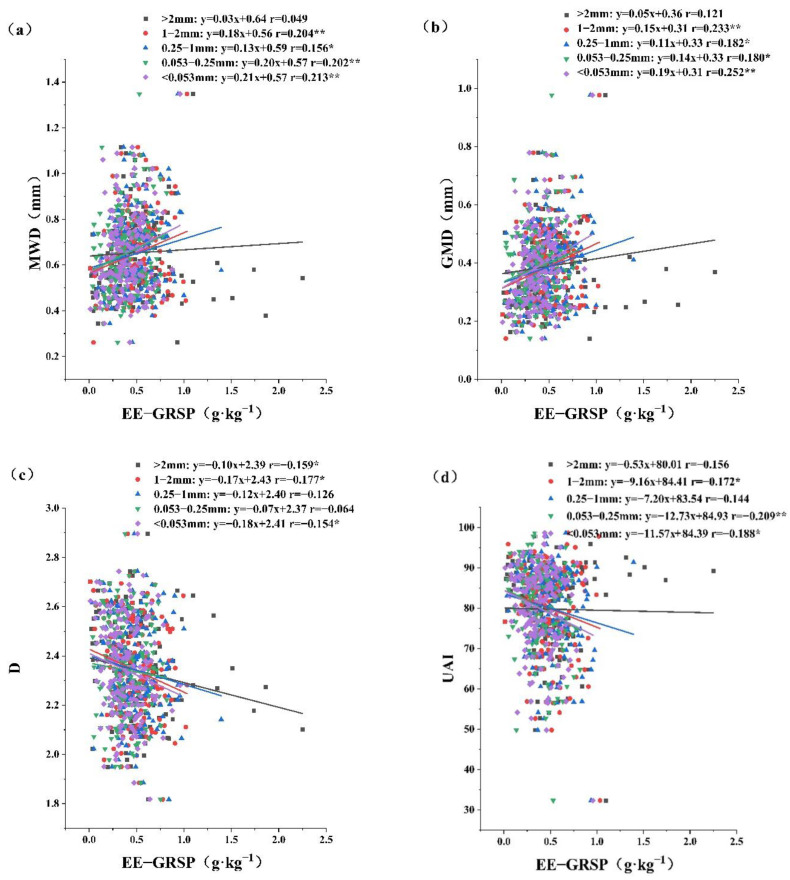

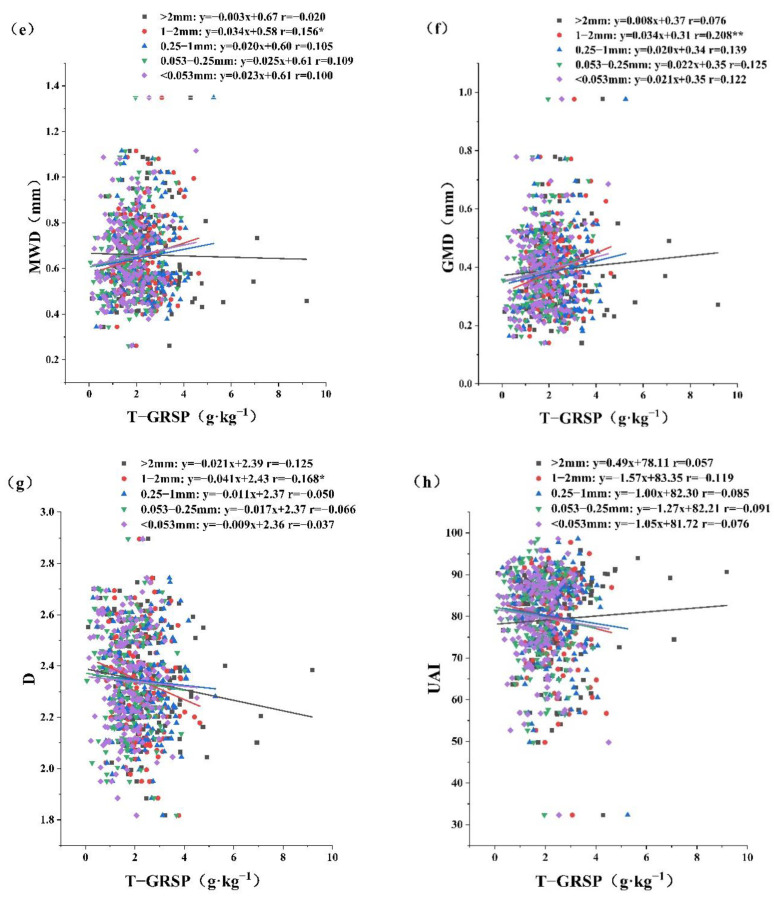
Correlations between GRSP and aggregate stability for different aggregates. Note: *: *p* < 0.05, **: *p* < 0.01. Easily extractable glomalin-related soil protein (EE-GRSP), total glomalin-related soil protein (T-GRSP), mean weight diameter (MWD), geometric mean diameter (GMD), fractal dimension (D), unstable aggregate index (UAI). Relationships between EE-GRSP and MWD (**a**), GMD (**b**), D (**c**), UAI (**d**), and relationships between T-GRSP and MWD (**e**), GMD (**f**), D (**g**), UAI (**h**).

The correlations between GRSP and the different soil aggregate stability indicators are shown in Figure 3. The four aggregates of particles <2 mm showed significant positive correlations between EE-GRSP and MWD and GMD (*p* < 0.05); the correlation coefficient was the largest for the <0.053 mm particle size (Figure 3a,b). The most significant correlation between EE-GRSP and the D value was found for the 1–2 mm aggregate (*p* < 0.05) (Figure 3c). The EE-GRSP in the 1–2 mm, 0.053–0.25 mm and <0.053 mm particle size aggregates showed significant negative correlations with UAI (*p* < 0.05) (Figure 3d). Figure 3e–g showed that T-GRSP in the 1–2 mm aggregate was significantly correlated with MWD, GMD, and D (*p* < 0.05).

### 2.5. Relationships between GRSP and Soil Properties

The correlations between GRSP and soil properties are shown in Table 3. We found that soil pH was negatively correlated with EE-GRSP in the >2 mm, 1–2 mm, 0.053–0.25 mm and <0.053 mm aggregates (*p* < 0.05). There was a significant negative correlation between bulk density and GRSP (T-GRSP and EE-GRSP) for the < 2 mm aggregates (*p* < 0.05). Soil moisture was positively correlated with GRSP (T-GRSP and EE-GRSP) for the 0.053–0.25 mm aggregate (*p* < 0.05).

## 3. Discussion

### 3.1. Increasing Urbanization Intensity Significantly Reduced the GRSP Content in the Different Soil Aggregates

The variation in GRSP content in the different aggregates showed clear spatial heterogeneity related to the intensity of urbanization. Both the EE-GRSP and T-GRSP contents showed the highest values in areas with a low urbanization intensity. Meanwhile, except for the >2 mm aggregates, the EE-GRSP content of the other four soil aggregate size classes were significantly higher (22–46%) in low urbanization intensity sites than in high urbanization intensity sites, indicating that severe anthropogenic disturbance areas were not conducive to GRSP secretion and accumulation. The results of this study are consistent with the findings of Jin et al. [32], in which increasing urbanization intensity was found to be associated with significantly reduced GRSP content. When compared to the low urbanization intensity areas, the GRSP content was significantly less by ~40% in the 1–2 mm aggregate from a high urbanization intensity area, indicating that the 1–2 mm aggregate mainly contributed to the reduced values of GRSP. The results of this study indicated that the 1–2 mm aggregate was more sensitive to the storage of GRSP, and it was also more susceptible to anthropogenic interference. Xiao et al. [33] also found that the T-GRSP content in 1–2 mm grain size aggregates in less anthropogenically disturbed soils was significantly higher than that in more anthropogenically disturbed soils. The values for 1–2 mm aggregates are most sensitive to changes in external factors over a short period of time [34]. This phenomenon can be attributed to the fact that artificial disturbance destroys soil structure, leading to the loss of soil organic matter, whereas no disturbance promotes the maintenance of soil organic matter [26]. When comparing the contribution of EE-GRSP to the T-GRSP content in different soil aggregates, the EE-GRSP/T-GRSP ratio showed an increasing trend with increased levels of urbanization, indicating that the ratio was higher in the large anthropogenically disturbed soils. Human disturbance factors can reduce the richness and activity of AMF, decrease the production of GRSP, and accelerate the decomposition of GRSP [35]. The EE-GRSP is a fresh GRSP produced by the symbiotic fungi and is not yet tightly bound to the soil particles [36], while the T-GRSP is derived by conversion of EE-GRSP [37]. These results indicate that although both T-GRSP and EE-GRSP contents decrease in poor or degraded soil, the decrease of T-GRSP is more rapid than EE-GRSP, resulting in an increase in the ratio.

First, changes in soil properties are an important factor affecting changes in GRSP values [38]. Soil pH was negatively correlated with EE-GRSP values in most aggregates, suggesting pH was one of the key factors for affecting EE-GRSP secretion. Neutral to slightly acidic soils have been suggested to be more suitable for plant root development and soil microbial colonization, allowing for more efficient accumulation of GRSP [39,40]. Moreover, a significant negative correlation between soil bulk density and the GRSP content has been reported [39]. The higher soil bulk density and pH in the high urbanization intensity area in this study (Table 3) led to the low GRSP content. Second, from a carbon stock perspective, the soil nutrient cycle in urban green spaces is altered by human living factors [41]. Similar to the results of this study, the carbon content of soils in suburban areas was found to be significantly higher than that in urban areas [42], indicating that anthropogenic disturbance factors should not be neglected in regards to the variation in GRSP content. Therefore, the effect of urbanization on the GRSP content of different aggregates must be integrated with intricate environmental factors in cities.

### 3.2. Carbon Sequestration Potential of GRSP in Different Soil Aggregates during Urbanization

Soil aggregate formation is an important process for SOC stabilization, and GRSP, an important component of SOC, has been found to play a key role in soil aggregate formation and stability enhancement [24,43,44]. In the process of urbanization, soil structure is becoming poor due to the anthropogenic disturbances of construction, trampling, rolling, and pollution (industry and life) [31]. Interestingly, the SOC and GRSP contents almost always showed significant differences among areas of different urbanization intensities, that is, a low urbanization intensity area was significantly higher than the high urbanization intensity area, indicating that anthropogenic disturbances caused significant perturbations to the GRSP pools and soil carbon pools. Because of the intense human activities in urban areas, the material exchange between humans and soil is more frequent. Rapid urbanization increases the area of enclosed soil, such as buildings, roads, and city squares, which changes the supply and demand cycle of soil organic matter, and thus causes a change in the carbon cycle of urban ecosystems [45]. Soil with less human disturbance has more abundant carbon sources, resulting in a much higher SOC content. The AMF needs to rely on the photosynthetic products of host plants to provide carbon sources for their growth and development [46]. Organic matter provided by the host plants enters the soil in the form of GRSP, thus, GRSP plays an important role in SOC fixation and cycling [47]. However, the contributions of EE-GRSP and T-GRSP to SOC pools did not differ significantly among areas of different urbanization intensities, and significant differences were found in different sized soil aggregates. Therefore, the contribution of GRSP to the SOC pool may not be affected by urbanization in Nanchang.

For the different sized soil aggregates, the contribution of GRSP to SOC for the >0.25 mm aggregate was significantly lower than that of the <0.25 mm aggregate, indicating that the SOC of smaller aggregates was more stored in the soil as GRSP. This phenomenon may be attributed to the high clay content and large surface area of the micro-aggregates (<0.25 mm aggregate) [48]; the GRSP has strong adhesion and decomposition resistance, which can be combined into stable organic–inorganic compounds [49]. The SOC decomposition rate is slower because the larger aggregates (>0.25 mm) can provide better physical protection for the SOC [48]. In contrast, SOC decomposed more rapidly in smaller aggregates; therefore, more carbon existed in the soil in the form of GRSP, resulting in a higher GRSP/SOC value.

The results showed that GRSP content was significantly positively correlated with SOC in all the soil aggregates, which again verified that GRSP is an important component of the SOC pool. Studies have shown that GRSP can contribute about 27% of SOC; as soil humus only contributes about 8% of carbon, the carbon contribution capacity of GRSP is 2–24 times that of soil humus [29]. Although there are many differences between EE-GRSP and T-GRSP, both when correlated with SOC showed an increasing trend and then decreased with decreasing particle size. Jing et al. [50] found the strongest correlation between SOC and T-GRSP for the >0.25 mm aggregate. Wilson et al. [51] showed that only aggregates with a >0.25 mm particle size showed a significant reduction in SOC after fungicide application to the soil, whereas aggregates with a <0.25 mm particle size were not significantly affected, indicating that SOC in a larger particle size had a more significant correlation with GRSP. Based on the forementioned results, this study reveals the correlation between SOC and GRSP in the three larger particle aggregates of >2 mm, 1–2 mm, and 0.25–1 mm. The strongest correlation between SOC and GRSP in the larger particle aggregates was found for the 1–2 mm particle size, indicating that GRSP is most favorable for SOC fixation in the 1–2 mm aggregate. Studies have shown that soil microorganisms, especially fungi, play an important role in the formation and stability of large aggregates [52]. The AMF, as important microorganisms in most ecosystems, are beneficial to promote the SOC sequestration [22]. These findings provide a theoretical basis for further exploration.

### 3.3. Effect of GRSP on Soil Aggregate Stability during the Process of Urbanization

Interestingly, we found that the correlation coefficient between the EE-GRSP content and the aggregate stability index was much higher than that between T-GRSP and the aggregate stability index. It is now widely accepted that both the T-GRSP and EE-GRSP play important roles in the adhesion and stability of aggregates. Because the EE-GRSP has an efficient bonding efficiency and the T-GRSP has more stable properties [28], it was thought that the role of EE-GRSP might be more focused on the formation of soil aggregates, whereas that of T-GRSP is more focused on the structural stabilization of soil aggregates. However, our study found that the effect of the EE-GRSP on aggregate stability may be much greater than that of T-GRSP. The reason for this result is that the EE-GRSP is the most recently produced material and is more active, whereas the T-GRSP is relatively older and not easily produced [53].

In addition, a significant correlation between T-GRSP values and the aggregates was found only for the 1–2 mm grain size. A similar result was found by Wu et al. [54] under different inoculation conditions. This not only indicates that T-GRSP is not the main binder of aggregates [55], but also further suggests that the 1–2 mm grain size aggregates may be most closely correlated with GRSP in soils and are the best grain size for GRSP to contribute to the enhanced stability of aggregates.

## 4. Materials and Methods

### 4.1. Study Site Description

The study area was located in Nanchang City, in the northern part of Jiangxi Province, East China (28°09′–29°11′ N, 115°27′–116°35′ E), with an average elevation of ~30 m. It has a typical subtropical monsoon climate with four distinct seasons, abundant rainfall, and a total area of 7159 km^2^. Among the different land use patterns, vegetation coverage accounts for 4703.69 km^2^, or 63.37% of the total area of Nanchang. Impervious surface is the second largest land use type with 1553.68 km^2^, accounting for 20.93% of the total area, while water and naked land use types account for 13.08% and 2.63%, respectively [31].

### 4.2. Urbanization Intensity Description

**Figure 4:**
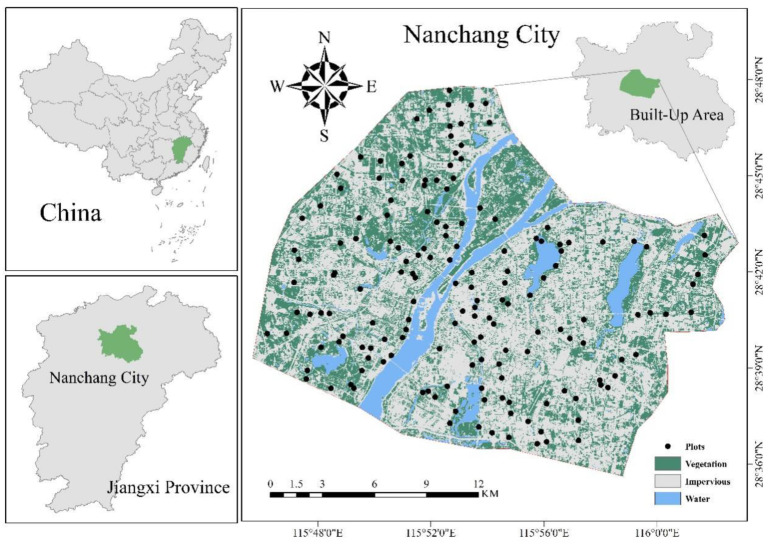
Overview of the Study Area.

The built-up area of Nanchang (505 km^2^) was used as the study area (Figure 4). The study area was divided into several 100 m × 100 m grids, and the proportion of impervious area was calculated for each grid. According to the proportion of impervious area, the grids were divided into three levels: low urbanization intensity (<50%), medium urbanization intensity (50% ≤ impervious area proportion < 80%), and high urbanization intensity (≥80%). Finally, 184 sampling plots (400 m^2^) from urban roadside forests, affiliated forests, landscape forests, and ecological and public welfare forests were set up in different urbanization areas. To reduce errors caused by plant species, forest plots were placed where the city’s official tree, camphor, was the dominant species, with a minimum spacing of 1 km between samples.

### 4.3. Soil Sampling

Soil sampling was conducted during the summer of 2020. Each sample plot was set up with five sampling points, and 0–20 cm column of soil was collected using a cutting ring (100 cm^3^). Soil from each sample plot was mixed and weighed immediately, and then taken back to the laboratory to air dry to a constant weight and this value recorded. The soil samples were divided into two parts after removal of impurities (plant roots, stones, plastics, plant litter, etc.): one for determination of soil physical and chemical properties [42,56] (Table 4), and the other for separation of soil aggregate fractions. The soil bulk density was calculated as the ratio of soil dry weight (105 °C) to soil volume. Soil (<2 mm) pH was determined using a soil-water ratio of 1:2.5, potentiometric method. Soil moisture content was determined using a drying method at 105 °C. The soil texture (<2 mm) was determined using a laser diffraction particle size analyzer (MS3000, Malvern, UK).

### 4.4. Soil Water Stable Aggregates Determination and Stability Index Calculation

Soil aggregates were separated by the wet sieve method [57]. Briefly, 100 g of air-dried soil samples were weighed to separate into five different particle size aggregates: >2 mm, 1–2 mm, 0.25–1 mm, 0.053–0.25 mm, and <0.053 mm. The soil aggregate stability was described using four indices: mean weight diameter (MWD), geometric mean diameter (GMD), unstable aggregate index (UAI), and fractal dimension (D). These indices were calculated using the following equations [58,59].

(1) $$\mathrm{MWD}=\underset{i=1}{\overset{n}{\sum }}{\overset{-}{x}}_{i}\omega_{i}$$

(2) $$\mathrm{GMD}=exp\left(\underset{i=1}{\overset{n}{\sum }}\omega_{i}\ln{\overset{-}{x}}_{i}\right)$$

where ${\overset{-}{x}}_{i}$ is the mean diameter of *i*th sieve and $\omega_{i}$ is the proportion of the total weight in the *i*th fraction.

(3) $$\mathrm{UAI}=\frac{W_{0}-W_{r>0.25}}{W_{0}}\times 100\%$$

where $W_{0}$ is the sum of the masses of each particle class of the soil; $W_{r>0.25}$ is the sum of the masses of particle size aggregates >0.25 mm after wet sieving.

Fractal dimension (D) was adopted from the soil particle fractal model proposed by Yang et al. [60].

(4) $$D=3-\frac{\mathrm{lg}\left(W_{i}/W_{0}\right)}{\mathrm{lg}\left({\overset{-}{d}}_{i}/{\overset{-}{d}}_{max}\right)}$$

where ${\overset{-}{d}}_{max}$ is the mean diameter of the largest particle size, *W* (δ<${\overset{-}{d}}_{i}$) is the accumulated mass of soil particles with a diameter less than ${\overset{-}{d}}_{i}$; $W_{0}$ is the sum of the mass of each particle size of the soil.

### 4.5. Soil Organic Carbon and Glomalin-Related Soil Protein Determination

The collected aggregates of each particle size were finely ground and passed through a 0.149 mm sieve. The SOC was determined using the potassium dichromate heating method [56]. The EE-GRSP and T-GRSP were extracted in accordance with the method described by Wright et al. [23,61]. Specifically, EE-GRSP was extracted using sodium citrate solution (20 mmol·L^−1^, pH = 7.0). Soil samples of 0.500 g and 4 mL of sodium citrate solution were mixed and put into an autoclave and sterilized at 121 ℃ for 30 min, followed by centrifugation at 4000 r·min^−1^ for 6 min in a high-speed centrifuge, and then the supernatant was collected. The T-GRSP was extracted using a sodium citrate solution of 50 mmol·L^−1^ (pH = 8.0) and an autoclaving time of 1 h. The above procedure was repeated until the supernatant no longer showed a typical red–brown color. Finally, the supernatant was colored using the Coomassie brilliant blue method, and the standard curve was plotted using bovine serum protein as the standard solution to calculate the EE-GRSP and T-GRSP contents.

### 4.6. Statistical Analysis

After testing the data for normal distribution, an ANOVA was performed using the Duncan’s multiple comparison method (SPSS 22) to explore the differences in the distribution and stability of aggregates of each particle size and the changes in SOC and GRSP content at sites of different urbanization intensities. Pearson’s correlation analysis was used to reveal the correlations among aggregate stability, SOC content, and GRSP content.

## 5. Conclusions

The GRSP participates in the formation of soil aggregates, and it also plays an important role in soil carbon pooling and cycling. An increasing urbanization intensity can significantly reduce the GRSP content of different soil aggregates. Although the carbon sequestration potential of GRSP in soil aggregates is apparently unrelated to urbanization, it is actually due to the degradation of soil quality caused by anthropogenic disturbance factors. This makes it more difficult to visualize the relationship of carbon sequestration potential of GRSP in soil aggregates with urbanization and this topic needs to be further explored. The EE-GRSP played a more critical role than the T-GRSP in enhancing soil aggregate stability. The GRSP is a component of the SOC pool and has a significant positive correlation, especially in the 1–2 mm grain size range. The T-GRSP also shows a significant contribution to the enhancement of aggregate stability in the 1–2 mm grain size range. Therefore, soil aggregates with a 1–2 mm particle size may be the best for GRSP-contributed aggregate stability enhancement in urban soils. This provides a new research direction for future in-depth investigations. This study fully reveals and explores the potential of GRSP to enhance soil aggregate formation and carbon storage in the context of rapid urbanization.

## Figures and Tables

**Table 1:** Percentage of soil water stable aggregates at different urbanization intensities.

Urbanization Intensity	>2 mm	1–2 mm	0.25–1 mm	0.053–0.25 mm	<0.053 mm
Low	10.11 ± 0.97 Ca	10.99 ± 0.51 Ca	43.03 ± 1.06 Aa	16.86 ± 0.65 Bb	19.01 ± 1.06 Ba
Medium	8.93 ± 0.73 Ca	9.88 ± 0.51 Ca	41.79 ± 1.31 Aa	19.46 ± 0.83 Ba	19.94 ± 1.22 Ba
High	8.86 ± 1.14 Ca	11.02 ± 0.78 Ca	40.85 ± 1.38 Aa	20.40 ± 1.15 Ba	18.87 ± 1.66 Ba

Note: Data (mean ± SE). Different capital letters indicate significant differences within the row (*p* < 0.05), while different lowercase letters indicate significant differences within the column (*p* < 0.05).

**Table 2:** Stability differences of the soil water stable aggregates at different urbanization intensities.

Urbanization Intensity	MWD	GMD	D	UAI
Low	0.67 ± 0.02 a	0.40 ± 0.02 a	2.33 ± 0.02 a	78.90 ± 1.31 a
Medium	0.63 ± 0.02 a	0.37 ± 0.01 a	2.36 ± 0.02 a	81.19 ± 1.06 a
High	0.64 ± 0.03 a	0.37 ± 0.02 a	2.33 ± 0.03 a	80.11 ± 1.90 a

Note: Data (mean ± SE). Different lowercase letters indicate significant differences within the column (*p* < 0.05). Mean weight diameter (MWD), geometric mean diameter (GMD), fractal dimension (D), unstable aggregate index (UAI).

**Table 3:** Relationships between GRSP and soil properties in different soil aggregates.

Soil Aggregates		pH	Bulk Density	Soil Moisture
EE-GRSP	>2 mm	−0.15 *	−0.10	−0.10
	1–2 mm	−0.30 **	−0.34 **	0.08
	0.25–1 mm	−0.12	−0.28 **	0.09
	0.053–0.25 mm	−0.29 **	−0.33 **	0.15 *
	<0.053 mm	−0.25 **	−0.35 **	0.09
T-GRSP	>2 mm	0.01	−0.14	−0.04
	1–2 mm	−0.05	−0.21 **	0.06
	0.25–1 mm	0.02	−0.29 **	0.16 *
	0.053–0.25 mm	0.03	−0.21 **	0.18 *
	<0.053 mm	−0.04	−0.20 **	0.06

Note: *: *p* < 0.05, **: *p* < 0.01. Easily extractable glomalin-related soil protein (EE-GRSP), total glomalin-related soil protein (T-GRSP).

**Table 4:** Soil physical and chemical properties in different urbanization intensity.

Urbanization Intensity	Bulk Density (g/cm^3^)	pH	Moisture Content (%)	Soil Texture
Low	1.31 ± 0.01	6.76 ± 0.09	21.77 ± 0.77	Silty loam
Medium	1.35 ± 0.01	7.09 ± 0.09	19.17 ± 0.81	Sandy loam
High	1.39 ± 0.02	7.29 ± 0.16	18.14 ± 0.89	Sandy loam

Note: Data (means ± SE).

## Data Availability

Data available on request due to restrictions eg privacy or ethical.
